# Access to eye care and support services among adults from minority ethnic communities living with visual impairment in the United Kingdom

**DOI:** 10.3389/fpubh.2023.1277519

**Published:** 2024-01-08

**Authors:** Nikki Heinze, Lee Jones

**Affiliations:** ^1^BRAVO VICTOR, Research, London, United Kingdom; ^2^UCL, Institute of Ophthalmology, London, United Kingdom

**Keywords:** visual impairment, sight loss, minority ethnic, BamE, service use, eye health, support, health inequalities scope statement

## Abstract

**Background:**

Despite an increased risk of certain eye conditions which can lead to visual impairment (V.I.), there is evidence of a greater delay to treatment-seeking among adults from minority ethnic communities (MEC). MEC adults may also be underrepresented on V.I. registers, within early intervention services, and among the beneficiaries of national V.I. charities. However, much of this evidence is outdated or anecdotal.

**Methods:**

This secondary analysis of V.I. Lives survey data explored use of eye health and support services and mobility aids among a matched control sample of 77 MEC and 77 adults aged 18 and over from white communities (WC). Participants were matched on age, gender, UK region and urban/rural setting. Additional subgroup analysis was conducted for Asian (*n* = 46) and black participants (*n* = 22).

**Results:**

There were no significant group differences in areas such as eye health service use, registration status, contact with charities, and level of practical support received. But MEC participants were significantly more likely than WC participants to have received direct payments from social services to cover their care needs, *Χ*^2^ (1, 154) = 8.27, *p* = 0.004, and to use apps on their mobile for mobility, *Χ*^2^ (1, 154) = 5.75, *p* = 0.017. In contrast, WC participants were significantly more likely to agree that they were getting the level of emotional support to get on with their life, *U* = 3,638, *p* = 0.010, to feel confident to ask their friends for support, *U* = 2,416, *p* = 0.040, and to have a guide dog for mobility, *Χ*^2^ (1, 154) = 3.62, *p* = 0.057, although the latter did not reach statistical significance. Within the MEC group, Asian participants were significantly more likely than black participants to use a long cane, *Χ*^2^ (1, 68) = 7.24, *p* = 0.007, but they were significantly less likely to agree that they had received the right level of support when they started to experience V.I., *U* = 236.5, *p* = 0.040.

**Conclusion:**

The preliminary findings suggests that there is scope to increase support provided by V.I. charities and the V.I. register, although, contrary to existing evidence, there were no statistically significant differences in eye health service use, registration status and use of wider support services. Further research is required to confirm these findings and explore reasons for differences.

## 1 Introduction

The number of people living with visual impairment (V.I.) in the UK is estimated to increase from around 2 million to approximately 4 million by 2050 (1). Data from the 2021 UK Census shows that minority ethnic communities (MEC) make up an increasing proportion of the population in England and Wales (2). People from certain MEC have been found to be at increased risk of V.I. (3, 4) and certain eye conditions which can result in V.I. relative to people from white communities (WC). For instance, people from Afro-Caribbean communities have been found to be at increased risk of primary open-angle glaucoma across all age groups (5, 6), while people from Asian communities may be at increased risk and earlier onset of diabetic retinopathy (7), including sight-threatening diabetic retinopathy (8), and cataract (9). As such, they are projected to make up an increasing proportion of adults living with V.I. in the UK (10).

In the UK, the eyecare pathway usually commences with an initial appointment with a community optometrist or general practitioner (GP), who may refer patients for diagnosis to secondary or tertiary eyecare. Diagnosis by an ophthalmologist may be followed by a treatment or monitoring phase, which requires regular eye appointments usually with an ophthalmologist or specialist community optometrist. Despite the increased risk of V.I. and eye disease, there is evidence of a greater delay to treatment-seeking among MEC adults. Research in the field of diabetic eye disease found that there was a significantly longer delay in attending an appointment at an eye clinic following a referral among Asian people than Black and White people and there was a significantly longer delay from referral (and also first appointment) to receiving treatment for their diabetic eye disease among Black people than Asian and White people (11). This suggests that people from Black communities may be waiting longer for treatment, while people from Asian communities may be waiting longer to receive a diagnosis. Research with adults from Indian communities explored barriers to use of further eyecare services (12). These included dissatisfaction with prior health service experiences, including long waiting times, limited awareness of how to access services, limited acceptance of Western medicine resulting in treatment being sought abroad, lack of time, health not being seen as a priority, and language. In addition, unhelpful perceptions of sight loss and limited awareness and understanding of eye conditions may result in fear and treatment-seeking only once symptoms were no longer manageable. For instance, cataracts were thought to require an undetermined period of maturing or ‘*ripening*’ until the condition was no longer manageable before treatment was thought necessary. Similarly, due to perceptions of glaucoma as being associated with aging, the perceived risk of experiencing glaucoma was low among younger African-Caribbean adults (13).

V.I. has been associated with a negative impact on activities of daily living (14, 15), participation in sports and leisure activities (16, 17), quality of life, mental health outcomes and social functioning (18–23). A recent rapid evidence review found limited research on the impact of V.I. among different ethnic communities in the UK (24). People who have been diagnosed with a V.I. have several support options. In the UK, individuals with a moderate or severe V.I. can be registered as sight impaired (partially sighted) or severely sight impaired (blind). To join the register, people need to first be certified as sight impaired (partially sighted) or severely sight impaired (blind) by an ophthalmologist (25). Certification is based on best-corrected visual acuity and visual field (26). A copy of the certificate is sent to the patient’s local council which will contact individuals with the offer to join the register (27). Registering a V.I. with local social services has a number of practical benefits including access to a needs assessment and appropriate support to remain independent, as well as financial concessions on transport, television and health services. There is evidence that MEC adults (28), including Asian adults (7) are less likely to be registered than WC adults. This may be due to limited awareness and knowledge of the benefits and registration process resulting from inhibition, particularly among older adults, and/or communication difficulties with clinicians (28). Some eye clinics provide early interventions services such as the Eye Clinic Liaison Officer (ECLO). These can offer advice and support relating to hospital appointments, registering a V.I., benefits, education, employment, housing, low vision aids or training, travel and social networks, and refer or signpost patients and their families to social services, sight loss charities or support groups following a diagnosis of irreversible sight loss (29, 30). However, Slade (29) reports that only 3.6% of service users were from Black and other minority ethnic communities while 96.4% were from White communities, suggesting that MEC may be underrepresented in early intervention services. Finally, sight loss charities can provide vision rehabilitation and mental health support following diagnosis and beyond. But anecdotal evidence suggests that MEC adults may be less aware of support services and the benefits associated with them (31, 32). This may result in unmet needs. For instance, qualitative research with Somali refugees who had V.I. found that language barriers may prevent these individuals from accessing statutory and other support resulting in unmet needs for support with activities of daily living including housework, and social isolation where participants were unable to leave the house without support (33). Anecdotal evidence also suggests that MEC adults may prefer to receive support from V.I. groups specifically for their ethnic community rather than national sight loss charities (31). This may be due to the cultural appropriateness of these services. Johnson and Morjaria-Keval (32) recommend hiring MEC staff, providing information materials in different languages, drawing on community partners to disseminate information, and providing funding and resources to community partners who offer support services within their communities, among other. To overcome negative experiences, they recommend building relationships and providing a continuous service. Overall, this suggests that service use may be lower among MEC, although the evidence is limited and/or anecdotal. There is further anecdotal evidence that guide dogs may not be an acceptable mobility aid among Somali community (34) and Afro-Caribbean communities (35). This raises important questions around the acceptability of available mobility aids among different communities, which is yet to be explored.

The current article forms part of a series of articles which explore the wider experiences of MEC adults living with V.I. in the United Kingdom. This article explores awareness and use of health and support services among a sample of MEC adults, including those from Asian and Black communities.

## 2 Materials and methods

This article uses secondary data collected in the V I Lives survey (36), a telephone survey of people with V.I. commissioned by the Royal National Institute of the Blind (RNIB), the Thomas Pocklington Trust (TPT) and Guide Dogs for the Blind Association (Guide Dogs).

### 2.1 Data and sample

Details of the survey and sample have been described elsewhere (36, 37). Briefly, participants were recruited through a healthcare database, local and national charities, social media, and radio adverts. People without V.I. and those who did not speak English were excluded. Wave 1 of fieldwork took place from 17 December 2019 to 23 March 2020. Wave 2 of fieldwork ran from 14 August 2020 to 2 November 2020.

To control for the unequal subgroup sample sizes and statistically significant differences between MEC and WC participants, a matched control sample was drawn using R (38). WC participants were matched to MEC participants based on their age, gender, region and whether participant lived in rural areas vs. towns.

### 2.2 Materials

A questionnaire was developed for the survey covering a wide range of topics. V.I. severity was assessed using a participant’s self-reported registration status (sight impaired/partially sighted or severely sight impaired/blind). Where participants were not registered, V.I. severity was determined with a set of questions adapted from the Life Opportunities Survey. These assessed the extent to which participant’s had difficulties seeing ordinary newsprint at arm’s length (near vision), the face of someone across the room *ca.* 4 m/12 ft. away (distance vision), and people or things in the periphery of their vision (peripheral vision). Finally, participants were asked about their legal driving status. Those who reported wearing glasses or contact lenses, were asked to rate near, distance and peripheral vision difficulties with glasses or contact lenses. Individual cases were reviewed and discussed by a panel to ensure they met inclusion criteria and to resolve inconsistent responses.

*Ethnicity*: participants were asked to indicate how they would describe their ethnic background from a list including *white British, white other, mixed/multiple ethnic groups, Asian/Asian British, black/African/Caribbean/black British and other ethnic group*.

*Use of health services*: a single question asked participants if they remembered roughly when they last visited an eye clinic.

*Registration status*: participants were asked if, as far as they knew, they were registered as severely sight impaired or sight impaired. Further response categories included *Registered but do not know which category* and *Not registered*.

*Awareness and use of charities:* three questions asked participants to select from lists (1) the charities for people with V.I. they had heard of (2) the charities they had contacted or had contact with, and (3) the other charities that they had contacted or had contact with in relation to their V.I.

*Support received*: One question explored which types of support services participants had ever received in relation to their V.I. The extent to which participants’ support needs had been met was explored in three questions which asked participants to what extent they agreed or disagreed that they got the level of practical support (a) and emotional support (b) they needed to get on with their life, and that they had received the right level of support when they started to experience sight impairment (c). Participants who were born with V.I. were not asked the last question. Participants were also asked to indicate how confident they were in asking for support from personal networks (family, friends, neighbors, network of visually impaired people they knew), authorities (government agencies, local councils), social workers/NHS services, wider support (charities, volunteers who visit people in their home) and religious groups.

*Use of mobility aids*: participants were asked whether they *normally used any kind of mobility aid, such as guide dog or long cane*. Interviewers then coded all aids used from a list. Those who did not have a guide dog or dual assistance dog were asked to indicate why this was the case.

### 2.3 Data analysis

Although the survey was not specifically designed to compare subgroups and despite low subsample sizes, the data analysis explored status within and group differences between MEC (*n* = 77) and WC participants (*n* = 77), as well as between the two largest MEC subgroups, participants from Asian (*n* = 46) and Black communities (*n* = 22), to gain a preliminary insight into service use among these groups.

For all variables, response distributions were calculated as counts (*n*) and proportions (%) for each subgroup. Subgroup analysis was performed using Mann–Whitney *U* tests for ordinal and chi-square tests, or Fisher’s exact test where assumptions of expected cell counts were violated, for categorical variables. Data analysis was conducted using SPSS (39) except for Fishers’ exact tests which were conducted using R.

## 3 Results

Table 1 provides an overview of participant characteristics by subgroup. Mean age was similar across all groups. A majority across all groups resided in a big town or city, specifically London, and were in employment. Around half of Asian, MEC and WC participants were female, and a majority were educated to degree-level and categorized as having severe V.I. In comparison, the proportion of females was higher among Black participants (59.1%), who were also more likely to be educated to Master’s level and to be categorized as having moderate V.I.

**Table 1:** Participant characteristics by subgroup.

	Asian (*n* = 46)	Black (*n* = 22)	MEC (*n* = 77)	WC (*n* = 77)
	% (*n*)	% (*n*)	% (*n*)	% (*n*)
Age	*U* = 500.5, *p* = 0.942		*U* = 2919.5, *p* = 0.871	
*M (SD)*	40.17 (14.61)	39.18 (14.70)	40.78 (15.58)	41.09 (15.62)
Range	18–74	18–75	18–85	18–85
Gender	*Χ*^2^ (1, 68) = 0.49, *p* = 0.482		*Χ*^2^ (1, 154) = 0.00, *p* = 1.00	
Female	50.0 (23)	59.1 (13)	51.9 (40)	51.9 (40)
Male	50.0 (23)	40.9 (9)	48.1 (37)	48.1 (37)
Region	*p* = 0.789		*p* = 0.344	
London	41.3 (19)	59.1 (13)	44.2 (34)	31.2 (24)
South East	4.3 (2)	9.1 (2)	6.5 (5)	2.6 (2)
South West	6.5 (3)	−	5.2 (4)	3.9 (3)
East of England	6.5 (3)	4.5 (1)	5.2 (4)	2.6 (2)
East Midlands	2.2 (1)	4.5 (1)	3.9 (3)	5.2 (4)
West Midlands	6.5 (3)	−	5.2 (4)	2.6 (2)
North East	−	−	−	5.2 (4)
North West	17.4 (8)	9.1 (2)	13.0 (10)	23.4 (18)
Yorkshire & the Humber	4.3 (2)	4.5 (1)	3.9 (3)	3.9 (3)
Scotland	4.3 (2)	9.1 (2)	7.8 (6)	9.1 (7)
Wales	4.3 (2)	−	3.9 (3)	7.8 (6)
Northern Ireland	2.2 (1)	−	1.3 (1)	2.6 (2)
Setting	*p* = 0.234		*Χ*^2^ (2, 154) = 4.68, *p* = 0.097	
City/big town	67.4 (31)	77.3 (17)	67.5 (52)	55.8 (43)
Small town	26.1 (12)	9.1 (2)	22.1 (17)	37.7 (29)
Rural area	6.5 (3)	13.6 (3)	10.4 (8)	6.5 (5)
Education^1^	*U* = 518, *p* = 0.086		*U* = 2,794, *p* = 0.397	
No formal qualifications	−	−	−	5.2 (4)
GCSE/O-Level	15.2 (7)	4.5 (1)	11.7 (9)	14.3 (11)
A-Level/Advanced Higher	15.2 (7)	9.1 (2)	15.6 (12)	18.2 (14)
Apprenticeship, vocational, NVQ or HND	17.4 (8)	18.2 (4)	16.9 (13)	11.7 (9)
Undergraduate degree	30.4 (14)	22.7 (5)	27.3 (21)	31.2 (24)
Masters, PhD	15.2 (7)	31.8 (7)	18.2 (14)	16.9 (13)
Non-UK qualifications	4.3 (2)	−	3.9 (3)	−
Other	2.2 (1)	13.6 (3)	6.5 (5)	2.6 (2)
Employment^2^	*p* = 0.771		*Χ*^2^ (4, 154) = 0.33, *p* = 0.988	
Employed (including part-time)	41.3 (19)	54.5 (12)	42.9 (33)	40.3 (31)
Self-employed	8.7 (4)	4.5 (1)	6.5 (5)	5.2 (4)
Unemployed	19.6 (9)	9.1 (2)	14.3 (11)	14.3 (11)
Retired	6.5 (3)	9.1 (2)	10.4 (8)	11.7 (9)
Other^2^	23.9 (11)	22.7 (5)	26.0 (20)	28.6 (22)
V.I. severity^3^	*U* = 552.5, *p* = 0.516		*U* = 2.951, *p* = 0.922	
Severe	41.3 (19)	31.8 (7)	39.0 (30)	44.2 (34)
Moderate	34.8 (16)	40.9 (9)	35.1 (27)	23.4 (18)
Mild	23.9 (11)	27.3 (6)	26.0 (20)	31.2 (24)
Could not be classified	−	−	−	1.3 (1)

1 Statistical analysis excludes *Non-UK qualifications and Other*.

2 Due to expected frequencies of less than 5 in 5 cells (27.8%), the categories *Looking after family/home, Student, Long-term sick/disabled and Unpaid work (e.g., volunteering, intern, work experiences)* were collapsed into the *Other* category for the statistical analysis.

3 Statistical analysis excludes *Could not be classified*.

MEC, Minority ethnic communities (excluding white minorities); WC, White communities (including white minorities).

Statistically significant results are shown in bold. Results for Fisher’s exact test are shown as *p*-values only.

### 3.1 Eye health services

There were no statistically significant differences between MEC and WC participants, *U* = 2877.5, *p* = 0.801, nor between Asian and Black participants, *U* = 481.5, *p* = 0.898, in the use of eye health services (Table 2). A slightly lower proportion of WC (53.4%) than MEC participants (57.3%) attended an eye clinic in the last year. Similar proportions of Asian (57.8%) and Black participants (57.1%) visited an eye clinic within the last 12 months, with around a third in both groups attending an eye clinic in the last 6 months. Two Black participants reported that they had never visited an eye clinic. These had mild and moderate V.I., keratoconus/corneal dystrophy and nystagmus, and neither had registered their V.I.

**Table 2:** Eye care service use, registration status and type of support received, by subgroup.

	Asian (*n* = 46)	Black (*n* = 22)		MEC (*n* = 77)	WC (*n* = 77)	
	**% (*n*)**	**% (*n*)**		**% (*n*)**	**% (*n*)**	
Last visit to eye clinic	*U* = 481.5, *p* = 0.898			*U* = 2877.5, *p* = 0.801		
Has never visited an eye clinic	−	9.5 (2)		2.7 (2)	−	
More than 5 years ago	8.9 (4)	−		9.3 (7)	9.3 (7)	
3–5 years	11.1 (5)	4.8 (1)		8.0 (6)	12.0 (9)	
1–2 years	22.2 (10)	28.6 (6)		22.7 (17)	25.3 (19)	
6–11 months	26.7 (12)	23.8 (5)		25.3 (19)	22.7 (17)	
Less than 6 months ago	31.1 (14)	33.3 (7)		32.0 (24)	30.7 (23)	
Registration status	*Χ*^2^ (1, 68) = 0.09, *p* = 0.763^1^			*Χ*^2^ (1, 154) = 0.47, *p* = 0.491^1^		
Registered severely sight impaired	39.1 (18)	31.8 (7)		36.4 (28)	41.6 (32)	
Registered partially sight impaired	30.4 (14)	36.4 (8)		31.2 (24)	19.5 (15)	
Registered but do not know category	2.2 (1)	−		2.6 (2)	3.9 (3)	
Not registered	28.3 (13)	31.8 (7)		29.9 (23)	35.1 (27)	
Type of support received			***Χ***^**2**^**(1, 68)**			***Χ*** ^**2**^ **(1, 154)**
None of these	17.4 (8)	13.6 (3)	*p* = 1.00	15.6 (12)	27.3 (21)	3.12, *p* = 0.077
ECLO	28.3 (13)	31.8 (7)	0.09, *p* = 0.763	27.3 (21)	26.0 (20)	0.03, *p* = 0.855
Rehabilitation service from LA	37.0 (17)	22.7 (5)	1.38, *p* = 0.241	31.2 (24)	24.7 (19)	0.81, *p* = 0.369
RNIB helpline/specialist advice services	43.5 (20)	45.5 (10)	0.02, *p* = 0.878	46.8 (36)	36.4 (28)	1.71, *p* = 0.191
A guide dog/other services from GD	19.6 (9)	4.5 (1)	*p* = 0.149	15.6 (12)	24.7 (19)	1.98, *p* = 0.159
Personal care/support from social services	15.2 (7)	13.6 (3)	*p* = 1.00	16.9 (13)	23.4 (18)	1.01, *p* = 0.315
Direct payments from social services to cover my care needs	21.7 (10)	13.6 (3)	*p* = 0.524	20.8 (16)	5.2 (4)	**8.27, *p* = 0.004**
Assessment/advice from low vision clinic	50.0 (23)	68.2 (15)	2.00, *p* = 0.158	51.9 (40)	51.9 (40)	0.00, *p* = 1.00
Other	4.3 (2)	4.5 (1)	*p* = 1.00	3.9 (3)	2.6 (2)	*p* = 1.00

1 For the purpose of statistical comparison the three categories relating to registration (as severely sight impaired, partially sight impaired, and do not know category) were combined. Subgroup analysis explored associations between groups and being or not being registered.

MEC = Minority ethnic communities, WC=White communities, ECLO = Eye clinic liaison officer, LA = Local authority, RNIB = Royal National Institute of the Blind, GD = Guide Dogs for the Blind. Statistically significant results are shown in bold. Results of Fisher’s exact tests are shown as *p*-values only.

### 3.2 Registration status

There were no statistically significant associations between ethnicity and being registered or not registered, neither when comparing Asian and Black, nor when comparing MEC and WC participants (Table 2). Being unregistered was most common among WC (35.1%) followed by Black (31.8%) and Asian participants (28.3%). In addition, three WC and one Asian participant were registered but did not know in which category. A majority of unregistered Asian, Black and WC participants had mild V.I. (84.6, 85.7 and 84.6% respectively). The primary cause of V.I. among unregistered Asian patients was keratoconus/corneal dystrophy (30.8%), while unregistered Black and WC participants primarily had “other”, unlisted conditions (28.6 and 37.0% respectively).

### 3.3 Support received

**Figure 1:**
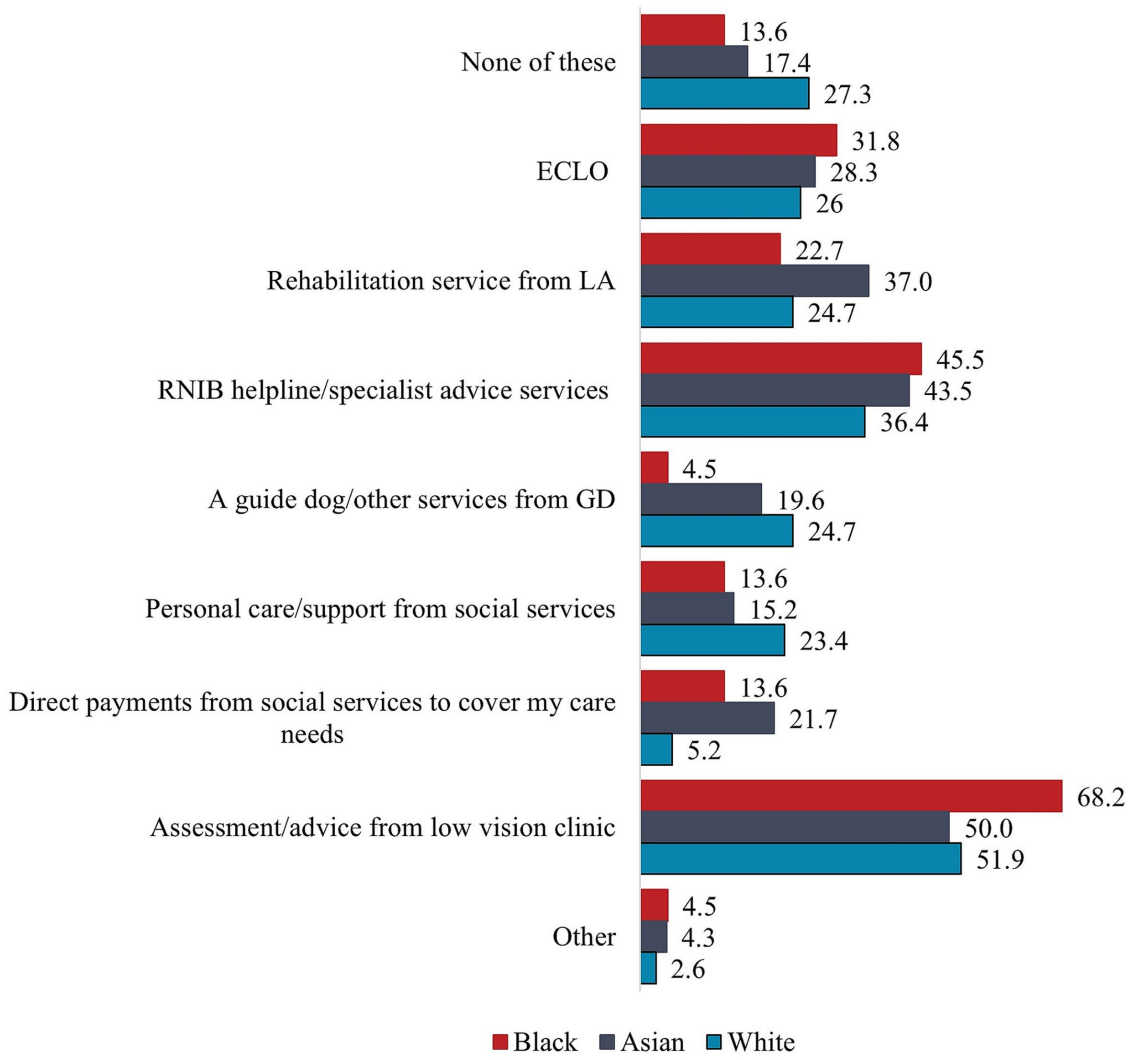
Support received by subgroup.

The survey assessed the types of support received, the adequacy of emotional, practical and initial support, as well as confidence in asking different sources for support. In terms of types of support received, MEC participants were four times more likely than WC participants to have received direct payments from social services to cover their care needs, 20.8% vs. 5.2%, *Χ*^2^ (1, 154) = 8.27, *p* = 0.004, Cramer’s V = 0.232. There were no further statistically significant associations between ethnicity and different support services available to people with V.I., neither for Asian and Black participants, nor for MEC and WC participants (Table 2). Although not statistically significant, WC participants (27.3%) were more likely to have received none of the listed support than MEC (15.6%), including Asian (17.4%) and Black participants (13.6%), but they were more likely than the MEC groups to have received a guide dog or other services from Guide Dogs as well as personal care and support from social services. All four WC participants who had been referred by Guide Dogs reported having received a guide dog or other support from Guide Dogs. In contrast, Black participants were more likely than other groups to have received support from a low vision clinic, the RNIB helpline or other specialist advice services and ECLOs, while Asian participants were more likely than other groups to have received rehabilitation services from their local authority and direct payments from social services (Figure 1). Assessment and advice from a low vision clinic followed by support from the RNIB helpline or other specialist advice services were the most common types of support received by participants in all groups. Half of Asian and WC participants and over two thirds of Black participants had received this.

In terms of the extent to which the support received was adequate, MEC participants were significantly less likely to agree that they were getting the level of emotional support to get on with their life, *U* = 3,638, *p* = 0.010. Eight in ten WC participants agreed with this compared to just over six in ten MEC participants (Table 3). Although not statistically significant, MEC participants were also less likely to agree that they got the level of practical support they needed to get on with their life but slightly more likely to agree that they got the right level of support when they started to experience V.I. While similar levels of Asian and Black participants agreed that they received sufficient emotional support (63.0% vs. 68.2% respectively), 54.6% of Black participants agreed that they received sufficient practical support compared to 67.4% of Asian participants, but 45.5% of Black participants agreed strongly with this compared to just 26.1% of Asian participants. At the other end of the scale, 21.7% of Asian participants disagreed that they received sufficient practical support compared to 36.4% of Black participants, but none of the Black participants disagreed strongly compared to 6.5% of Asian participants. These differences were not statistically significant. However, Black participants were significantly more likely to agree and agree strongly than Asian participants that they had received the right level of support when they started to experience V.I., *U* = 236.5, *p* = 0.040. Overall, around a third of Asian and Black participants did not believe they had received the right support when they lost their sight, nor the practical or emotional support they needed at the time of the survey, although the proportion of Asian participants who did not receive sufficient emotional support was lower (21.7%).

**Table 3:** Extent to which support needs were met and sources of support, by subgroup.

	Asian (*n* = 46)	Black (*n* = 22)	MEC (*n* = 77)	WC (*n* = 77)
	% (*n*)	% (*n*)	% (*n*)	% (*n*)
I get the level of practical support I need to get on with my life	*U* = 486, *p* = 0.785		*U* = 3160.5, *p* = 0.457	
Strongly agree	26.1 (12)	45.5 (10)	35.1 (27)	33.8 (26)
Slightly agree	41.3 (19)	9.1 (2)	31.2 (24)	41.6 (32)
Neither agree nor disagree	10.9 (5)	9.1 (2)	9.1 (7)	11.7 (9)
Slightly disagree	15.2 (7)	36.4 (8)	20.8 (16)	10.4 (8)
Strongly disagree	6.5 (3)	−	3.9 (3)	2.6 (2)
I get the level of emotional support I need to get on with my life	*U* = 484.5, *p* = 0.770		***U* = 3,638, *p* = 0.010**	
Strongly agree	30.4 (14)	36.4 (8)	32.5 (25)	45.5 (35)
Slightly agree	32.6 (15)	31.8 (7)	29.9 (23)	35.1 (27)
Neither agree nor disagree	15.2 (7)	−	10.4 (8)	9.1 (7)
Slightly disagree	13.0 (6)	27.3 (6)	18.2 (14)	10.4 (8)
Strongly disagree	8.7 (4)	4.5 (1)	9.1 (7)	−
I received the right level of support when I started to experience V.I.	***U* = 236.5, *p* = 0.040** (*n* = 56)		*U* = 1,765, *p* = 0.903 (*n* = 120)	
Strongly agree	18.9 (7)	52.6 (10)	27.7 (18)	30.9 (17)
Slightly agree	29.7 (11)	15.8 (3)	26.2 (17)	16.4 (9)
Neither agree nor disagree	13.5 (5)	−	9.2 (6)	10.9 (6)
Slightly disagree	10.8 (4)	21.1 (4)	13.8 (9)	20.0 (11)
Strongly disagree	27.0 (10)	10.5 (2)	23.1 (15)	21.8 (12)
Confidence asking for support from…				
Family	*U* = 629, *p* = 0.061		*U* = 2,694, *p* = 0.249	
Very confident	54.3 (25)	77.3 (17)	59.7 (46)	68.8 (53)
Quite confident	32.6 (15)	18.2 (4)	27.3 (21)	20.8 (16)
Not very confident	2.2 (1)	4.5 (1)	6.5 (5)	6.5 (5)
Not at all confident	10.9 (5)	−	6.5 (5)	3.9 (3)
Friends	*U* = 541, *p* = 0.504		***U* = 2,416, *p* = 0.040**	
Very confident	40.0 (18)	45.5 (10)	39.5 (30)	55.8 (43)
Quite confident	42.2 (19)	45.5 (10)	47.4 (36)	36.4 (28)
Not very confident	13.3 (6)	4.5 (1)	9.2 (7)	5.2 (4)
Not at all confident	4.4 (2)	4.5 (1)	3.9 (3)	2.6 (2)
Neighbors	*U* = 556, *p* = 0.397		*U* = 2,834, *p* = 0.727	
Very confident	15.6 (7)	18.2 (4)	14.5 (11)	11.7 (9)
Quite confident	17.8 (8)	27.3 (6)	21.1 (16)	31.2 (24)
Not very confident	31.1 (14)	27.3 (6)	31.6 (24)	24.7 (19)
Not at all confident	35.6 (16)	27.3 (6)	32.9 (25)	32.5 (25)
Network of vision impaired people	*U* = 510, *p* = 0.712		*U* = 2,842, *p* = 0.792	
Very confident	31.8 (14)	40.9 (9)	31.1 (23)	29.3 (22)
Quite confident	27.3 (12)	18.2 (4)	29.7 (22)	26.7 (20)
Not very confident	13.6 (6)	13.6 (3)	12.2 (9)	18.7 (14)
Not at all confident	27.3 (12)	27.3 (6)	27.0 (20)	25.3 (19)
Local councils	*U* = 459, *p* = 0.522		*U* = 2,920, *p* = 0.867	
Very confident	21.7 (10)	18.2 (4)	18.2 (14)	13.0 (10)
Quite confident	17.4 (8)	22.7 (5)	19.5 (15)	31.2 (24)
Not very confident	39.1 (18)	22.7 (5)	33.8 (26)	27.3 (21)
Not at all confident	21.7 (10)	36.4 (8)	28.6 (22)	28.6 (22)
Government agencies	*U* = 545.5, *p* = 0.591		*U* = 3,174, *p* = 0.431	
Very confident	10.9 (5)	27.3 (6)	16.9 (13)	9.1 (7)
Quite confident	30.4 (14)	22.7 (5)	27.3 (21)	27.3 (21)
Not very confident	34.8 (16)	18.2 (4)	28.6 (22)	37.7 (29)
Not at all confident	23.9 (11)	31.8 (7)	27.3 (21)	26.0 (20)
Social workers/NHS services	*U* = 595, *p* = 0.222		*U* = 2,929, *p* = 0.872	
Very confident	19.6 (9)	31.8 (7)	23.4 (18)	20.0 (15)
Quite confident	39.1 (18)	40.9 (9)	40.3 (31)	42.7 (32)
Not very confident	23.9 (11)	13.6 (3)	18.2 (14)	22.7 (17)
Not at all confident	17.4 (8)	13.6 (3)	18.2 (14)	14.7 (11)
Charities	*U* = 443.5, *p* = 0.396		*U* = 2,927, *p* = 0.882	
Very confident	32.6 (15)	27.3 (6)	30.3 (23)	14.5 (11)
Quite confident	23.9 (11)	22.7 (5)	23.7 (18)	42.1 (32)
Not very confident	21.7 (10)	13.6 (3)	18.4 (14)	28.9 (22)
Not at all confident	21.7 (10)	36.4 (8)	27.6 (21)	14.5 (11)
Volunteers	*U* = 514.5, *p* = 0.785		*U* = 2725.5, *p* = 0.533	
Very confident	13.3 (6)	22.7 (5)	18.4 (14)	21.1 (16)
Quite confident	20.0 (9)	22.7 (5)	19.7 (15)	18.4 (14)
Not very confident	28.9 (13)	9.1 (2)	22.4 (17)	27.6 (21)
Not at all confident	37.8 (17)	45.5 (10)	39.5 (30)	32.9 (25)
Religious groups	*U* = 568.5, *p* = 0.302		*U* = 3199.5, *p* = 0.082	
Very confident	11.1 (5)	13.6 (3)	11.8 (9)	5.5 (4)
Quite confident	13.3 (6)	31.8 (7)	19.7 (15)	13.7 (10)
Not very confident	33.3 (15)	18.2 (4)	25.0 (19)	26.0 (19)
Not at all confident	42.2 (19)	36.4 (8)	43.4 (33)	54.8 (40)

MEC, Minority ethnic communities; WC, White communities. Statistically significant results are shown in bold.

**Figure 2:**
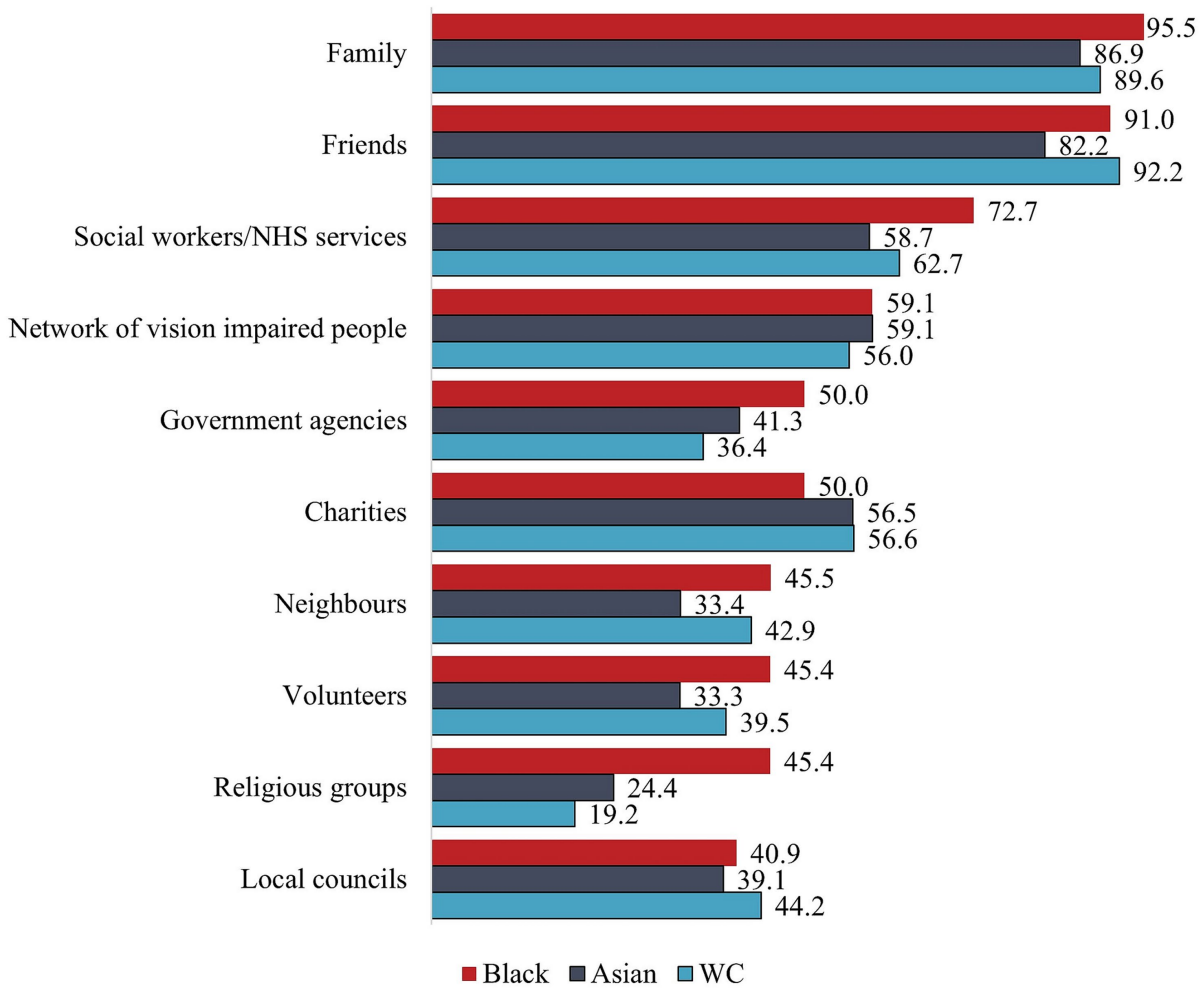
Feels very or quite confident to ask for support by subgroup.

WC participants were significantly more likely to feel confident to ask their friends for support than MEC participants, *U* = 2,416, *p* = 0.040 (Figure 2). There were no further statistically significant differences in the extent to which participants felt confident to ask a range of sources for support. While Black participants were more confident than Asian participants to ask family for support, and MEC participants were more confident than WC participants to ask religious groups for support, these differences did not reach statistical significance. Asian participants were generally less confident than Black participants in asking for support, except for their network of people with V.I. and charities. Black and Asian participants felt most comfortable asking family and friends for support. While Asian and WC participants felt least confident to ask religious groups for support, Black participants felt least confident asking local councils for support.

### 3.4 Awareness of and contact with charities

There were no statistically significant associations between ethnicity and contact with V.I. and other charities (Table 4). Support from V.I. charities was highest among WC and lowest among Black participants. Over a third of Black (36.4%) and Asian participants (34.8%) had not had any contact with any of the charities compared to 28.6% of WC participants. While the latter had had contact with an average of 1.78 (*SD* = 1.85, range 0–10) charities, this was slightly lower for Black (*M* = 1.73, *SD* = 2.33, range 0–10) and Asian participants (*M* = 1.50, *SD* = 1.63, range 0–5). Over half of participants in all groups had had contact with the RNIB, while contact with SeeAbility and Blind Veterans UK, who provide support for people with learning difficulties/autism and veterans with sight loss respectively, was low among all groups.

**Table 4:** Awareness and use of charity support, by subgroup.

	Asian (*n* = 46)	Black (*n* = 22)		MEC (*n* = 77)	WC (*n* = 77)	
	% (*n*)	% (*n*)	*Χ*^2^ (1, 68)	% (*n*)	% (*n*)	*Χ*^2^ (1, 154)
Awareness of V.I. charities						
RNIB	76.1 (35)	90.9 (20)	*p* = 0.197	81.8 (63)	90.9 (70)	2.70, *p* = 0.100
Guide Dogs	84.8 (39)	77.3 (17)	*p* = 0.505	83.1 (64)	90.9 (70)	2.07, *p* = 0.150
TPT	30.4 (14)	18.2 (4)	1.15, *p* = 0.284	26.0 (20)	20.8 (16)	0.58, *p* = 0.446
BVUK	37.0 (17)	50.0 (11)	1.05, *p* = 0.307	42.9 (33)	48.1 (37)	0.42, *p* = 0.517
Henshaws	23.9 (11)	18.2 (4)	*p* = 0.758	20.8 (16)	33.8 (26)	3.27, *p* = 0.070
Macular Society	34.8 (16)	18.2 (4)	1.98, *p* = 0.160	29.9 (23)	50.6 (39)	**6.91, *p* = 0.009**
Retina UK	34.8 (16)	31.8 (7)	0.06, *p* = 0.809	32.5 (25)	29.9 (23)	0.12, *p* = 0.728
Royal Society for Blind Children	50.0 (23)	59.1 (13)	0.49, *p* = 0.482	53.2 (41)	57.1 (44)	0.24, *p* = 0.627
SeeAbility	10.9 (5)	27.3 (6)	*p* = 0.156	14.3 (11)	26.0 (20)	3.27, *p* = 0.070
Sense	32.6 (15)	50.0 (11)	1.91, *p* = 0.167	37.7 (29)	50.6 (39)	2.63, *p* = 0.105
Local V.I. charity in the area	32.6 (15)	18.2 (4)	1.54, *p* = 0.215	27.3 (21)	40.3 (31)	2.90, *p* = 0.088
None of these	2.2 (1)	4.5 (1)	*p* = 0.546	2.6 (2)	1.3 (1)	*p* = 1.00
Contact with V.I. charities						
RNIB	58.7 (27)	54.5 (12)	0.10, *p* = 0.746	58.4 (45)	59.7 (46)	0.03, *p* = 0.870
Guide Dogs	26.1 (12)	18.2 (4)	0.52, *p* = 0.472	23.4 (18)	35.1 (27)	2.54, *p* = 0.111
TPT	13.0 (6)	13.6 (3)	*p* = 1.00	11.7 (9)	9.1 (7)	0.28, *p* = 0.597
BVUK	−	4.5 (1)	*p* = 0.324	1.3 (1)	5.2 (4)	*p* = 0.367
Henshaws	6.5 (3)	9.1 (2)	*p* = 0.656	6.5 (5)	9.1 (7)	0.36, *p* = 0.548
Macular Society	2.2 (1)	9.1 (2)	*p* = 0.243	3.9 (3)	9.1 (7)	1.71, *p* = 0.191
Retina UK	4.3 (2)	9.1 (2)	*p* = 0.590	5.2 (4)	7.8 (6)	0.43, *p* = 0.513
Royal Society for Blind Children	2.2 (1)	9.1 (2)	*p* = 0.243	5.2 (4)	5.2 (4)	*p* = 1.00
SeeAbility	−	9.1 (2)	*p* = 0.101	2.6 (2)	3.9 (3)	*p* = 1.00
Sense	6.5 (3)	18.2 (4)	*p* = 0.202	10.4 (8)	6.5 (5)	0.76, *p* = 0.385
Local V.I. charity in the area	28.3 (13)	18.2 (4)	0.81, *p* = 0.369	22.1 (17)	26.0 (20)	0.32, *p* = 0.572
None of these	34.8 (16)	36.4 (8)	0.02, *p* = 0.898	33.8 (26)	28.6 (22)	0.48, *p* = 0.486
Contact with other charities						
Age UK	4.3 (2)	-	*p* = 1.00	2.6 (2)	2.6 (2)	*p* = 1.00
Alzheimer’s Society	−	−	−	−	−	−
Arthritis Care	−	−	−	−	−	−
Arthritis UK	−	−	−	−	−	−
Diabetes UK	4.3 (2)	−	*p* = 1.00	2.6 (2)	1.3 (1)	*p* = 1.00
Stroke UK	−	4.5 (1)	*p* = 0.324	1.3 (1)	1.3 (1)	*p* = 1.00
Versus Arthritis	−	−	−	−	−	−
Other	15.2 (7)	13.6 (3)	*p* = 1.00	15.6 (12)	15.6 (12)	*p* = 1.00
Have never contacted charities	56.5 (26)	63.6 (14)	0.31, *p* = 0.577	55.8 (43)	44.2 (34)	2.10, *p* = 0.147

MEC, Minority ethnic communities; WC, White communities; RNIB, Royal National Institute for Blind People; TPT, Thomas Pocklington Trust; BVUK, Blind Veterans UK. Statistically significant results are shown in bold. Results of Fisher’s exact tests are shown as *p*-values only.

A similar pattern emerged for non-V.I. charities. Contact with these was again highest among WC and lowest among Black participants. Almost two thirds of Black (63.6%) and over half of Asian participants (56.5%) had not had contact with other charities. Most participants in all groups had contact with ‘other’, unlisted charities. A small number of participants also had contact with Stroke UK (Black and WC participants), Age UK and Diabetes UK (Asian and WC participants respectively).

Awareness of V.I. charities was higher than actual contact, but there were also no statistically significant associations between ethnicity and awareness of different sight loss charities, except for a significantly higher awareness of the Macular Society among WC (50.6%) compared to MEC participants (29.9%), *Χ*^2^ (1, 154) = 6.91, *p* = 0.009, Cramer’s V = 0.212. RNIB and Guide Dogs were the best-known charities among all groups, with Black participants being more aware of RNIB and Asian participants being more aware of Guide Dogs. The least-known charity among Asian participants was SeeAbility (10.9%), while comparatively lower proportions of Black participants (18.2% respectively) were aware of TPT, Henshaws, Macular Society and local V.I. charities. TPT was also the least-known charity among WC participants (20.8%). Except for RNIB, Guide Dogs and the Royal Society for Blind Children, fewer than half of Asian participants were aware of the other V.I. charities listed. While at least half of Black participants were also aware of Blind Veterans UK and Sense, awareness of the other charities was lower than for Asian participants except for SeeAbility. One Asian, Black and WC participant, respectively, had never heard of any of the V.I. charities listed.

### 3.5 Use of mobility aids

A majority in all groups reported that they did not use any mobility aids (Table 5), although the same proportions of Asian participants reported using no aids as the long cane. Asian participants were over four times more likely to use a long cane (the most common aid among this group), *Χ*^2^ (1, 68) = 7.24, *p* = 0.007, Cramer’s V = 0.326, while Black participants were around three times more likely to use voice activated sat nav or GPS, but this did not reach statistical significance, *p* = 0.066. Voice-activated sat nav/GPS and apps on mobile phones were the most common mobility aids used by Black participants, while none used symbol canes or had a guide dog.

**Table 5:** Use of mobility aids and reasons for not having a guide dog, by subgroup.

	Asian (*n* = 46)	Black (*n* = 22)		MEC (*n* = 77)	WC (*n* = 77)	
	% (*n*)	% (*n*)		% (*n*)	% (*n*)	
Use of mobility aids			*Χ*^2^ (1, 68)			*Χ*^2^ (1, 154)
None	41.3 (19)	45.5 (10)	0.10, *p* = 0.746	40.3 (31)	50.6 (39)	1.68, *p* = 0.195
Long cane	41.3 (19)	9.1 (2)	**7.24, *p* = 0.007**	31.2 (24)	28.6 (22)	0.12, *p* = 0.725
Symbol cane	6.5 (3)	−	*p* = 0.546	3.9 (3)	3.9 (3)	*p* = 1.00
Guide cane	8.7 (4)	4.5 (1)	*p* = 1.00	7.8 (6)	2.6 (2)	*p* = 0.276
Walking stick	2.2 (1)	9.1 (2)	*p* = 0.243	3.9 (3)	3.9 (3)	*p* = 1.00
Walking frame	−	4.5 (1)	*p* = 0.324	1.3 (1)	−	*p* = 1.00
Guide dog/dual assistance dog	6.5 (3)	−	*p* = 0.546	5.2 (4)	14.3 (11)	3.62, *p* = 0.057
Voice activated Sat Nav/GPS	8.7 (4)	27.3 (6)	*p* = 0.066	13.0 (10)	5.2 (4)	2.83, *p* = 0.093
Technological enabled aids	4.3 (2)	4.5 (1)	*p* = 1.00	3.9 (3)	−	*p* = 0.245
Apps on mobile phone	17.4 (8)	27.3 (6)	*p* = 0.356	19.5 (15)	6.5 (5)	**5.75, *p* = 0.017**
Specific device for people with V.I.	6.5 (3)	4.5 (1)	*p* = 1.00	5.2 (4)	2.6 (2)	*p* = 0.681
Other	2.2 (1)	13.6 (3)	*p* = 0.096	6.5 (5)	2.6 (2)	*p* = 0.442
Why no Guide Dog			*Χ*^2^ (1, 65)			*Χ*^2^ (1, 139)
Have to be totally blind	2.3 (1)	9.1 (2)	*p* = 0.263	5.5 (4)	3.0 (2)	*p* = 0.683
V.I. is not serious enough	46.5 (20)	68.2 (15)	2.75, *p* = 0.097	52.1 (38)	57.6 (38)	0.43, *p* = 0.514
Do not like dogs	7.0 (3)	9.1 (2)	*p* = 1.00	6.8 (5)	3.0 (2)	*p* = 0.445
Shops/restaurants/venues refuse entry	4.7 (2)	4.5 (1)	*p* = 1.00	4.1 (3)	3.0 (2)	*p* = 1.00
Taxi drivers will not accept dogs	−	−	−	−	3.0 (2)	*p* = 0.224
Other people’s attitudes toward dogs	−	−	−	−	−	−
On waiting list for a dog	2.3 (1)	−	*p* = 1.00	2.7 (2)	3.0 (2)	*p* = 1.00
Insufficient mobility to need a guide dog	9.3 (4)	13.6 (3)	*p* = 0.681	11.0 (8)	−	***p* = 0.007**
Religious beliefs	2.3 (1)	−	*p* = 1.00	1.4 (1)	−	*p* = 1.00
Too much work and responsibility	16.3 (7)	13.6 (3)	*p* = 1.00	15.1 (11)	3.0 (2)	**5.93, *p* = 0.015**
Other	44.2 (19)	31.8 (7)	0.93, *p* = 0.335	39.7 (29)	34.8 (23)	0.35, *p* = 0.553

MEC, Minority ethnic communities; WC, White communities. Statistically significant results are shown in bold. Results of Fisher’s exact tests are shown as *p*-values only.

MEC participants were more likely than WC participants to use aids, but this was not statistically significant. MEC participants were three times more likely to use apps on their mobile, *Χ*^2^ (1, 154) = 5.75, *p* = 0.017, Cramer’s V = 0.193, while WC participants were just under three times more likely to have a guide dog,this did not reach statistical significance, *p* = 0.057. When asked why they did not have a guide dog, MEC participants were five times more likely to give too much work and responsibility as reasons, Χ^2^ (1, 139) = 5.93, *p* = 0.015, Cramer’s V = 0.206. In addition, 11.0% said they did not having sufficient mobility to need a guide dog, but none of the WC participants gave this as a reason.

A higher proportion of Black (68.2%) than Asian participants (46.5%) reported that their V.I. was not serious enough to need a guide dog, but this did not reach statistical significance, *p* = 0.097. The latter was the most common reason given among all groups. Only one person in the Asian group gave religious beliefs as a reason for not having a guide dog.

## 4 Discussion

This article provides a preliminary insight into awareness and use of V.I. services among a sample of MEC, including Asian and Black, participants.

Overall, there were few statistically significant differences between Black and Asian, and a matched control sample of MEC and WC participants in the support they had received. Among these, MEC participants were significantly more likely than WC participants to have received direct payments from social services to cover their care needs, but they were less likely to be aware of the Macular Society, agree that they got the level of emotional support they needed to get on with their life, and feel confident in asking friends for support. Within the MEC group, Black participants were significantly more likely than Asian participants to agree that they had received the right level of support when they started to experience V.I. Although neither was statistically significant, WC participants were more likely to agree that they received the practical support they needed, but they were least likely to agree that they had received the right level of support when they developed their V.I. There were further statistically significant differences between groups in the mobility aids they used: despite previous findings from qualitative research conducted in the UK that older MEC adults were less likely to have up-to-date technological devices (55), MEC participants in this sample were three times more likely to use apps on their mobile than WC participants, who, in turn, were just under three times more likely to have a guide dog. MEC participants were significantly more likely to give too much work and responsibility and not having sufficient mobility as reasons for not having a guide dog than WC participants. The lower uptake of guide dogs among MEC, particularly Black participants, is perhaps not surprising considering previous findings that guide dogs may not be appropriate among Afro-Caribbean (35) including Somali (34) communities. It has been suggested that this is due to religious or cultural beliefs which perceived dogs as wild animals rather than pets (33), but only one participant in the Asian group gave religious beliefs as a reason. Within the MEC group, Asian participants were over four times more likely than Black participants to use a long cane and, although not statistically significant, Black participants were around three times more likely to use voice activated sat nav or GPS. The comparatively higher use of technology versus mechanical mobility aids among Black participants reflects previous findings from the US which showed that use of assistive technologies (AT) was higher among African American (40, 41), but lower among Asian adults (41) compared to European American adults with disabilities. Indeed, African American adults with disabilities were 29% more likely to use AT than their European American counterparts (40). In contrast, Hispanic and African Americans were less likely to receive AT services than European Americans in the context of vocational rehabilitation (42). It is unclear to what extent these findings apply to the UK context, because research exploring ethnic group differences in the use of AT in the UK and among adults with V.I. is missing. This would be an interesting and important area of future research, first, to confirm if there are indeed ethnic group differences in AT and app usage, and second, to explore potential reasons for these. One reason for the higher use of apps among the Black participants in this particular sample may relate to preferences for more discreet AT. For example, other types of mobility assistance such as guide dogs and long canes were avoided due to their distinct association with V.I., whereas apps may be more convenient and easier to conceal. Invisible aids may be felt to be more appropriate among communities with particularly negative perceptions of people with V.I. which may make them reluctant to identify as having V.I. (13, 33). Indeed, none of the Black participants had a guide dog or used a symbol cane, which, contrary to the long cane, serves to notify others that the person has a V.I. rather than for mobility. Despite the preference for technological aids among Black participants, improving functioning and availability of technology was a bigger priority for Asian than Black participants in this sample (43). This may reflect a lack of awareness of available aids, suggesting that Asian communities may benefit from information and training about the different apps and technology that is available to support people living with V.I.

There were no statistically significant differences between groups in the last time they had visited an eye clinic nor in registration status. Contrary to existing evidence (7, 28), WC participants were slightly more likely to be unregistered (35.1%) than Black (31.8%) and Asian participants (28.3%). Although around 8 in 10 unregistered participants in all three groups had mild V.I. and may therefore not be eligible for registration, around 14% of unregistered participants in all groups had moderate or severe V.I.: of the 7 unregistered Black participants one was categorized as having moderate V.I., of the 13 unregistered Asian participants two were categorized as having moderate and two as having severe V.I., and of the 27 unregistered WC participants two were categorized as having moderate and two as having severe V.I. V.I. severity in this sample was assessed using self-reported registration status, near, distance and peripheral vision difficulties and driving status, rather than objective measures of vision status. It is possible that some participants were miscategorized, but there were no differences between groups in V.I. severity. It is unclear then if the higher proportion being unregistered among WC participants reflects an uptake of registrations among MEC adults or drop-off in registrations among WC adults since Pardhan and Mahomed (7) published their findings in 2002 and Barry and Murray (28) in 2005. Neither article provides proportions, but Barry and Murray (28) report that MEC adults were more than three times more likely to be unregistered (OR: 3.23, 95% CI: 1.56–6.65). Alternatively, the findings may reflect selection bias among MEC participants in this sample, whereby those who are in contact with services, including the V.I. register, and better supported were more likely to take part in the research. Considering the benefits associated with registration, it is important that evidence relating to uptake of registration among different communities is updated in a larger sample.

Existing evidence also suggests that MEC adults may be underrepresented among early intervention services such as ECLOs (29). However, MEC participants, particularly those from Black communities, were slightly more likely to have received support from ECLOs than WC participants in this sample, although this was not statistically significant. MEC participants were also more likely to have received some form of support, the most common among all groups being assessment and advice from a low vision clinic followed by support from specialist advice services including the RNIB helpline. The prevalence of support received from the RNIB hotline and similar specialist advice services may be expected considering that a proportion of the sample was recruited from lists provided by the RNIB. While all three Black participants referred by the RNIB had received this type of support, 8 of the 10 Asian participants and only 10 of the 18 WC participants referred by the RNIB reported that they had received support through the RNIB hotline or similar service.

The RNIB was also the best known V.I. charity. Although anecdotal evidence suggests that MEC adults may prefer to seek support from V.I. groups run by members of their community rather than national sight loss charities (31), there were no statistically significant differences for any of the charities listed including local V.I. charities in the area. Patterns of prevalence of contact with charities were similar for MEC and WC participants, though the proportion who have not had any contact with any of the V.I. charities listed was slightly higher among MEC than WC participants: over a third of Asian and Black participants have not had contact with any V.I. charities compared to over a quarter of WC participants. It is possible that this finding reflects a greater reluctance to approach charities for support. Indeed, the proportion of participants who were *not at all confident* in asking charities for support was much lower among WC than Asian and Black participants, but equal proportions of Asian and WC participants felt at least *quite confident* in asking charities for support. In contrast, half of Black participants felt *not very* or *not at all confident* in asking charities for support. Participants from Black communities also felt least confident asking their local council for support. This is of concern because it may impact on accessing vision rehabilitation, and emotional, financial and wider support available to this group. In addition, Asian participants, who were least likely to seek support from most sources, were less likely than the other groups to feel confident asking social and NHS services for support. This may impact on seeking an early diagnosis and treatment. Social services are not only responsible for the local V.I. register but can provide a range of practical and financial support to help people remain independent. Despite a higher proportion of WC participants receiving personal care and support from social services, Asian participants were most likely to receive direct payments from social services to cover their care needs. The lack of confidence in contacting social and NHS services could therefore relate to difficulties associated with the payment process. A lack of social care may also impact on the wider family with family members having to take the place of carers. Future research may need to explore the reasons for the lower confidence in medical and social services observed among Asian participants and how this impacts on eye health, care received and the wider family. While participants across all three groups felt most confident asking family and friends for support, there is some evidence that members of the Somali communities, for instance, may be reluctant to ask family for support or feel more comfortable asking a daughter than a son for support (33). Future research may need to explore gender roles and cultural differences in the extent to which people feel confident in seeking support from family members. At least half of participants in all three groups felt *not very* or *not at all confident* asking government agencies, local councils, neighbors, volunteers, and religious groups for support. Participants from Black communities were around twice as likely to feel at least *quite confident* in asking religious group for support, with the proportion being lowest among WC participants. The survey did not explore participants’ religious beliefs and affiliation. It is therefore not possible to explore if confidence was tied to specific religious groups or strength of religious beliefs. This may be a useful area of research as it may be possible to work with religious groups to disseminate information relating to eye health and available support.

### 4.1 Limitations

There are a number of sample-related limitations. Despite the cultural diversity of groups that tend to be categorized as “Asian” (44), it was not possible to compare more granular subgroups due to already small sample sizes. The relatively small sample size particularly among MEC subgroups may have impacted statistical power, resulting in the few statistically significant differences found in this study. The sample also does not include non-English speakers who may be less likely to access services or have access to services that are not provided in their mother tongue and therefore be in particular need of support. Conversely, although the majority of the sample were recruited through the Acumen health database, a small number were referred through sight loss charities such as RNIB and Guide Dogs. The participants in this study may therefore have been better supported than the general V.I. population in the UK. V.I. status was assessed using self-report, which relies on an honest and realistic response, including for a more objective measure such as registration status. Two articles (45, 46) compared subjective and objective measures of V.I. Both found some mismatches, including an overidentification of V.I. using self-report (46), nonetheless both concluded that self-report was a reasonable indicator of V.I. in surveys. While a full ophthalmic assessment would therefore be preferrable, self-reported V.I. continues to be used in V.I. research (45, 47–51).

The survey explored a wide range of topics. As a results, certain areas such as eye health service use, satisfaction with different aspects of wider support and areas where support and services were perceived to be missing were underexplored or not explored at all. As a result, future research with a bigger, representative sample will need to confirm the validity of the current findings and explore service use, support needs as well as barriers and facilitators.

## 5 Conclusion

This article provides a preliminary insight into the use of V.I. services and support among a sample of MEC, including Asian and Black, adults. Contrary to existing evidence, there were no statistically significant differences in eye health service use, registration of a V.I. and use of wider support services. The findings suggests that there is scope to increase uptake of support, including support provided by V.I. charities and through the V.I. register. Further research is required to understand the reasons why MEC participants felt less confident than WC participants in asking charities and their local council for support. Similarly, cultural differences in the acceptability of and resulting preferences for different types of mobility aids warrant further exploration to ensure that appropriate support can be provided.

### Scope statement

Visual impairment (V.I.) has been associated with a negative impact on a wide range of life domains including activities of daily living, sports and leisure activities, social activities quality-of-life and mental health. There are various eye health and support services available to those living with V.I. in the UK. However, there is some evidence of inequalities relating to ethnicity and eye service use in the UK: adults from minority ethnic communities (MEC) may delay seeking a diagnosis or treatment, be underrepresented among services, which can provide vital support such as early intervention services, and the V.I. register, which can provide financial and functional support. However, much of the evidence requires confirmation or updating and/or relates to a BAME supergroup. This secondary analysis provides preliminary insights into the use of eye health and a range of support services among a sample of MEC, including Asian and Black, adults. Although there were few statistically significant differences, the findings provide practical insights into habits and patterns relating to service and mobility aid use among the different groups, highlighting, for instance, that there is scope to increase uptake of support provided by V.I. charities and through the V.I. register among MEC participants.

## Data availability statement

The original contributions presented in the study are included in the article/supplementary material, further inquiries can be directed to the corresponding author.

## Ethics statement

Ethical approval was not required for the study involving humans in accordance with the local legislation and institutional requirements. Written informed consent to participate in this study was not required from the participants or the participants’ legal guardians/next of kin in accordance with the national legislation and the institutional requirements.

## Author contributions

NH: Conceptualization, Data curation, Formal analysis, Methodology, Visualization, Writing – original draft. LJ: Methodology, Visualization, Writing – review & editing.
